# Supplementary material for: Long-Term Effects of Biliverdin Reductase a Deficiency in Ugt1−/− Mice: Impact on Redox Status and Metabolism
Source: Antioxidants (Basel). 2021 Dec 20;10(12):2029. doi: 10.3390/antiox10122029 (PMC8698966; doi:10.3390/antiox10122029)
Supplement: Supplementary file 1 [file antioxidants-10-02029-s001.zip › antioxidants-1437576-supplementary.pdf]

Long-Term Effects of Biliverdin Reductase a Deficiency in *Ugt1*$^{-/-}$ Mice: Impact on Redox Status and Metabolism

Giulia Bortolussi [1,†], Xiaoxia Shi [2,3,†], Lysbeth ten Bloemendaal [2], Bhaswati Banerjee [1], Dirk R. De Waart [2], Gabriele Baj [4], Weiyu Chen [5], Ronald P. Oude Elferink [2], Ulrich Beuers [2], Coen C. Paulusma [2], Roland Stocker [5], Andrés F. Muro [1,*] and Piter J. Bosma [2,*]

Supplemental material section

Supplemental Figures and Legends to Supplemental Figures

Table S1. Number of animals used in each of the Figures/Panels.

Figure	Parameter	wt	Ugt1-/-	Bvra-/-	DKO
**1A**	Survival Phototherapy	0	3	12	6
**1B**	Bilirubin phototherapy	3	3	8	3
**1C**	Survival normal light	0	4	10	9
**1D**	Bilirubin normal light	2	3	4	4
**2C**	Amniotic fluid	7	0	3	0
**2D**	Plasma embryos	7	0	5	0
**3A**	Plasma	9	10	11	9
**3B**	Bile	9	10	10	10
**3C**	Urine	9	3	4	4
**4A**	Reticuolcytes 3 months	10	0	10	10
**4B**	RBC 3 months	10	0	10	10
**4D**	RBC 9 months	9	8	7	10
**4E**	Reticuolcytes 9 months	9	8	7	10
**5B**	Non heme iron	9	11	10	10
**5C**	Spleen weight	11	10	11	10
**6A-D**	mRNA expression	9	14	11	10
**7A**	PPARα	9	10	11	10
**7B**	TG plasma	9	10	10	10
**7C**	Blood glucose	9	8	7	10
**S1B**	Weight curves	20	27	31	17
**S3A**	AST	9	10	11	10
**S3B**	ALT	9	10	11	10
**S3C**	Liver weight	11	10	11	10
**S4**	Hematological analysis - 3 months	10	9	10	10
**S4**	Hematological analysis - 9 months	9	8	7	10

**S7 A,B, and C**	Spleen and Liver	9	14	11	10
**S7C**	Liver	9	10	11	10
**S8A**	Tg in liver	7	10	8	9
**S8B,C and D**	PPARα responsive genes	9	10	11	10
**S9A**	Body weight Males	6	5	6	5
**S9A**	Body weight Females	5	5	5	5
**S9B**	Glucose Males	4	5	4	5
**S9B**	Glucose Females	5	3	3	5

Table S2. Sequence of the primers used to determine mRNA levels by qPCR.

Target	MGI/NCBI Gene	Sense	Antisense
Hmox1	96163/15368	5′-CTCGAATGAACACTCTGGAGAT-3′	5′-GCGGTGTCTGGGATGAGCTA-3′
Ppara	104740/19013	5′-CCCTGTTTGTGGCTGCTATAATTT-3′	5′-GGGAAGAGGAAGGTGTCATCTG-3′
Cpt1a	1098296/12894	5′-TGGCATCATCACTGGTGTGTT-3′	5′-GTCTAGGGTCCGATTGATCTTTG-3′
Fgf21	1861377/56636	5′-CCTCTAGGTTTCTTTGCCAACAG-3′	5′-AAGCTGCAGGCCTCAGGAT-3′
Gys2	2385254/232493	5′-CCAGCTTGACAAGTTCGACA-3′	5′-ATCAGGCTTCCTCTTCAGCA-3′
Gapdh	95640/14433	5′-GACAACTCATCAAGATTGTCAGCA-3′	5′-TTCATGAGCCCTTCCACAATG-3′
Nfe2l2	108420/18024	5’-TTCTTTCAGCAGCATCCTCTCCAC-3’	5’-ACAGCCTTCAATAGTCCCGTCCAG-3’
Nqo1	103187/1804	5’-GCTGCAGACCTGGTGATATT-3’	5’-ACTCTCTCAAACCAGCCTTT-3’
Gclm	104995/14630	5’-GACAAAACACAGTTGGAACAGC-3’	5’-CAGTCAAATCTGGTGGCATC-3’
Hamp	1933533/84506	5’-TTGCGATACCAATGCAGAAGA-3’	5’- GATGTGGCTCTAGGCTATGTT-3
Tnf	104798/21926	5’-TGGAACTGGCAGAAGAGGCACT-3	‘5’ CCATAGAACTGATGAGAGGGAGGC -3’
Il6	96559/16193	‘5’- GAGTTGTGCAATGGCAATTCTG-3’	5’- TGGTAGCATCCATCATTTCTTTGT-3

Table S3. Crossing-breeding of Bvra and Ugt1a deficient mice.

$Bvra^{+/-}$ x $Bvra^{-/-}$	Total number of pups born 9		
Genotype	Number expected	Number Observed	Statistics
$Bvra^{+/-}$	4.5	4	Chi-square df: 0.05247, 1<br>P value : 0.8188<br>N.S.
$Bvra^{-/-}$	4.5	5	

$Bvra^{+/-}Ugt1a^{+/-}$ x $Bvra^{-/-}Ugt1a^{-/-}$		Total number of pups born 65	
Genotype	Number expected	Number observed	Statistics
$Bvra^{+/-}$ $Ugt1a^{+/+}$	8	7	Chi-square df: 3.633, 5<br>P-Value: 0.6033<br>N.S.
$Bvra^{+/-}$ $Ugt1a^{+/-}$	16	18	
$Bvra^{+/-}$ $Ugt1a^{-/-}$	8	3	
$Bvra^{-/-}$ $Ugt1a^{+/+}$	8	10	
$Bvra^{-/-}$ $Ugt1a^{+/-}$	16	21	
$Bvra^{-/-}$ $Ugt1a^{-/-}$	8	6	

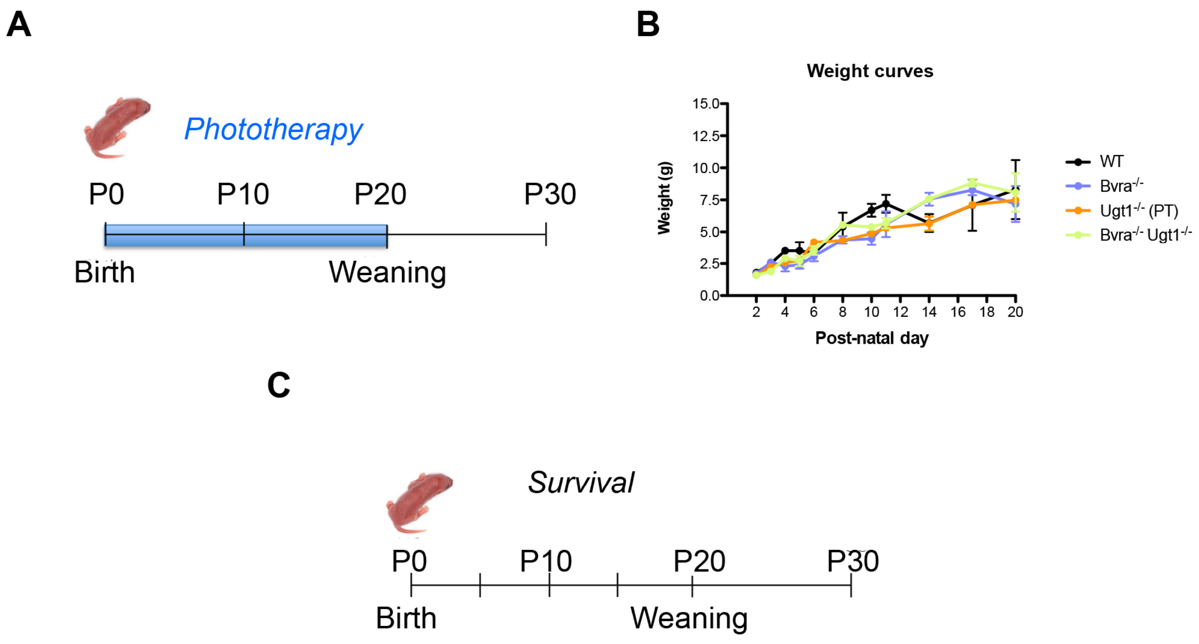

Figure S1. Experimental design and weight curve. A) Scheme of the experimental design. Newborn mice were housed exposing them to phototherapy (PT) for 20 days, a condition that results in survival of all Ugt1$^{-/-}$ mice. Mice were weaned at postnatal day 20 and survival was monitored up to postnatal day 30. B) Weight curve during early postnatal life (from P2 to P20, mixed genders). Two-way Anova. Interaction genotype-gender ***, P<0.0001; genotype, ***, P=0.0001; gender, ***, P=0.0001. Bonferroni post-test. WT vs. *Bvra*$^{-/-}$*,* ***, P<0.001 at P10, P11, P14, P20. WT vs. Ugt1$^{-/-}$, **, P<0.01 at P10; ***, P<0.001 at P11; *, P<0.05 at P20. WT vs. *Bvra*$^{-/-}$*Ugt1*$^{-/-}$*,* **, P<0.01 at P11; ***, P<0.001 at P14 and P17. *Bvra*$^{-/-}$ vs. *Ugt1*$^{-/-}$*,* **, P<0.01 at P14. *Bvra*$^{-/-}$ vs. *Bvra*$^{-/-}$*Ugt1*$^{-/-}$*,* *, P<0.05 at P20. Ugt1$^{-/-}$ vs. *Bvra*$^{-/-}$*Ugt1*$^{-/-}$*,* *, P<0.05 at P8; **, P<0.01 at P14 and P17; C) Scheme of the experimental design. Newborn mice were housed without exposing them to phototherapy (PT), a very severe condition that results in the early death of all *Ugt1*$^{-/-}$ mice. Mice were weaned at postnatal day 20 and survival was monitored up to postnatal day 30.

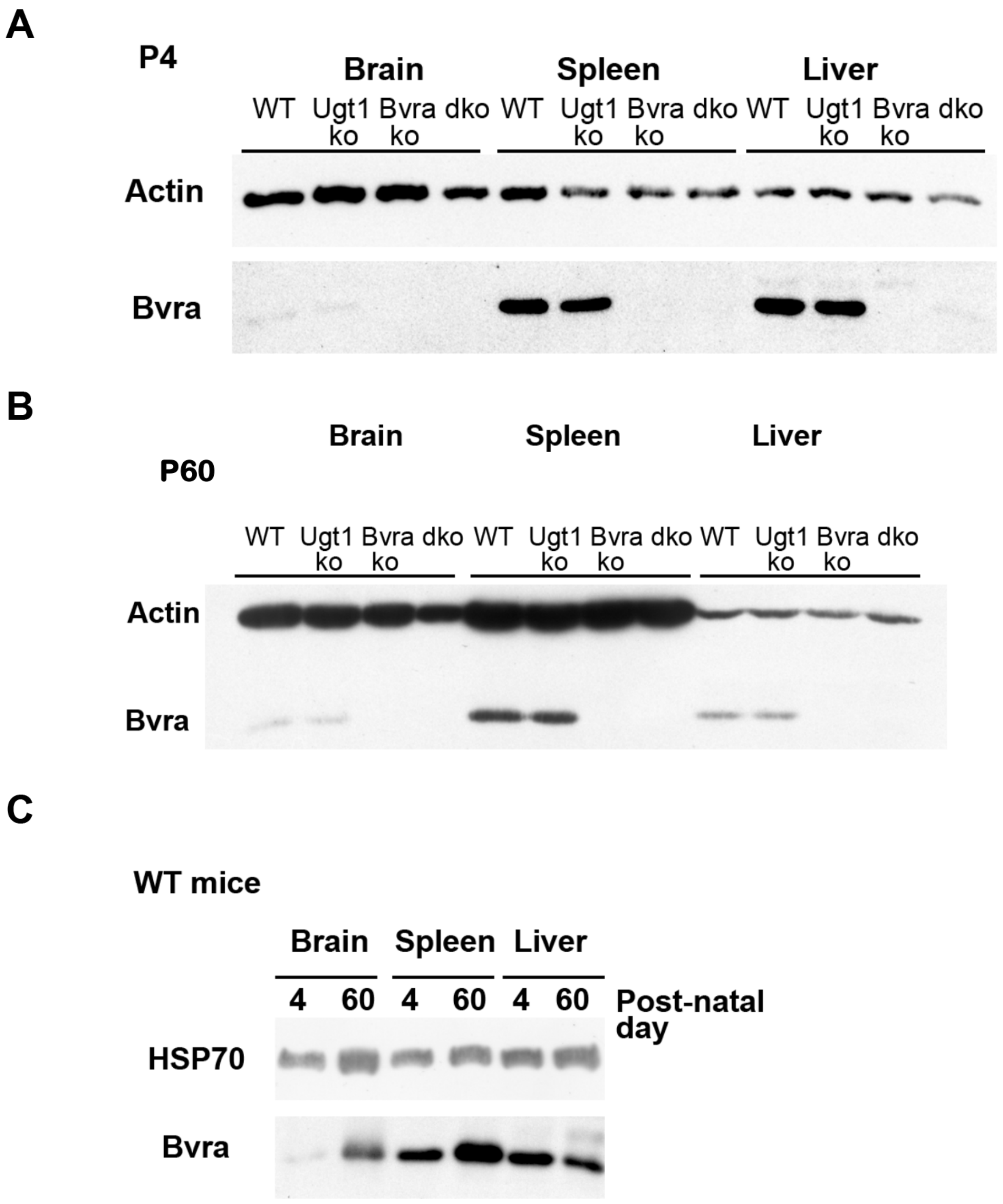

Figure S2. Bvra protein levels in P4 and P60 mice. A) Western blot analysis of brain, spleen and liver samples from P4 mice. Actin was used as loading control. Genotypes are indicated; B) Western blot analysis of brain, spleen and liver samples from P60 mice. Actin was used as loading control. C) Western blot of brain, spleen and liver from P4 and P60 WT mice. HSP70 was used as loading control.

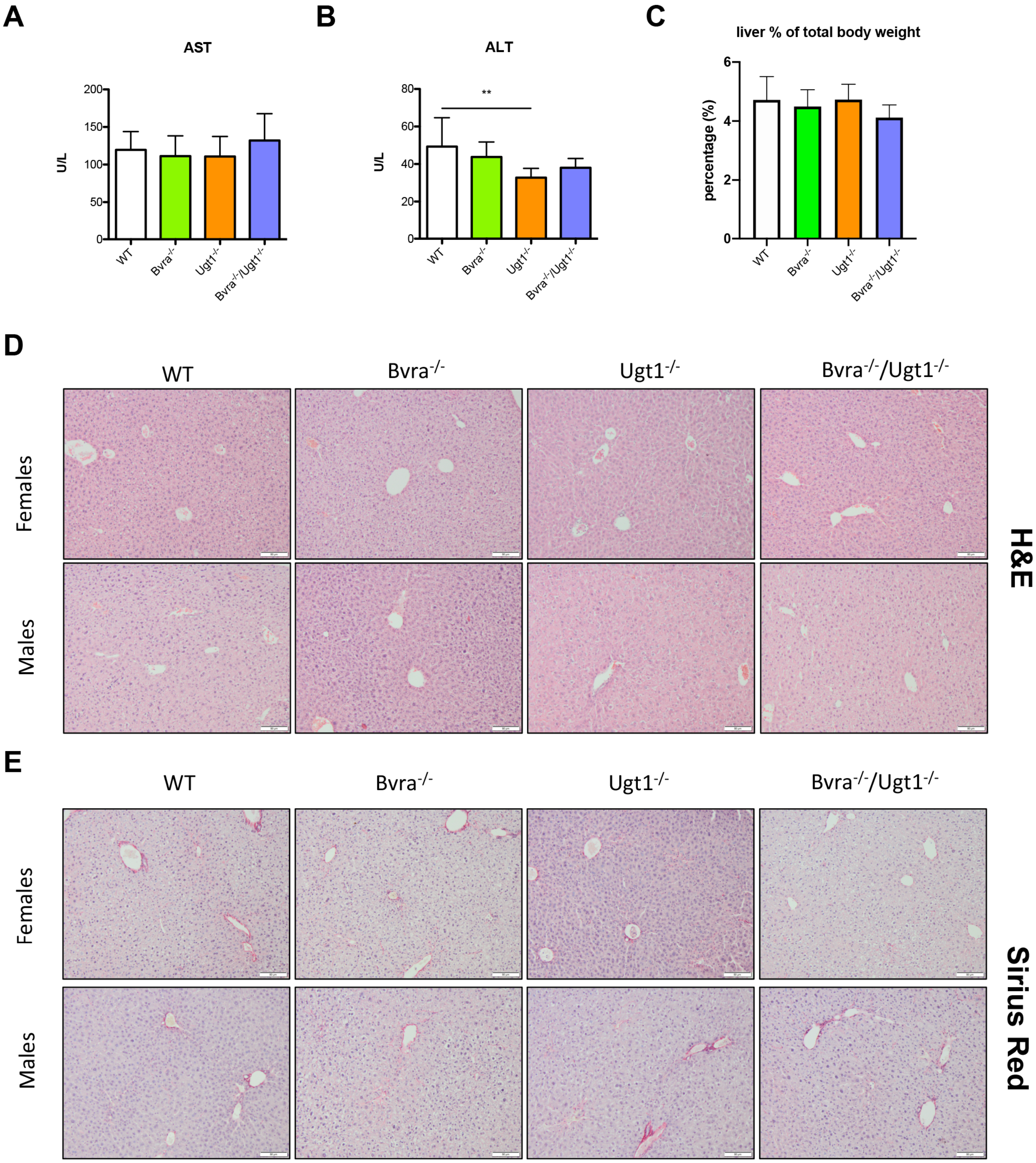

Figure S3. In 9 month old mice Bvra deficiency does not affect liver transaminases levels in plasma, nor liver histology. A) Aspartate transaminase levels in plasma. 1-way Anova, ns; B). Alanine transaminase level in plasma (ALT). 1-way Anova followed by Bonferroni post-hoc test, WT vs. *Bvra*$^{-/-}$*Ugt1*$^{-/-}$*,* ns. Data represent mean ± SD; C) Liver weight determination as percentage of body weight. 1-way Anova, P=0.0907, F=2.320, NS; D) Representative images of liver HE staining from 9 months old mice. E) Representative images of liver Sirius Red staining from 9 months old mice. Scale bar 50 µm.

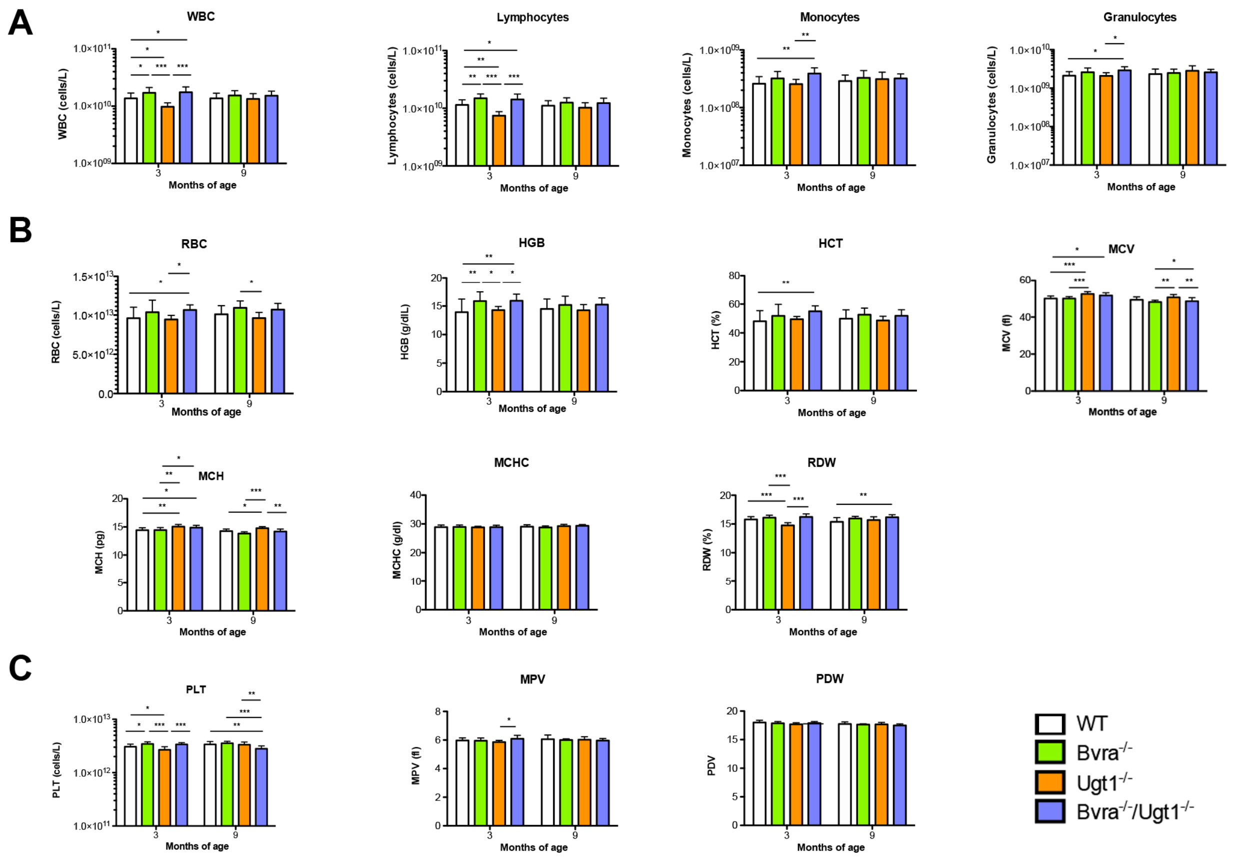

Figure S4. Whole blood hematological analysis. A) Data of the white cell compartment. B) Data of the red cell compartment. C) Platelet parameters. Data represent mean ± SD. 2-way Anova (genotype and time). Bonferroni post-test are indicated in the corresponding panels. *, p≤0.05, **, P≤0.01, ***, P≤0.001; ns, not significant.

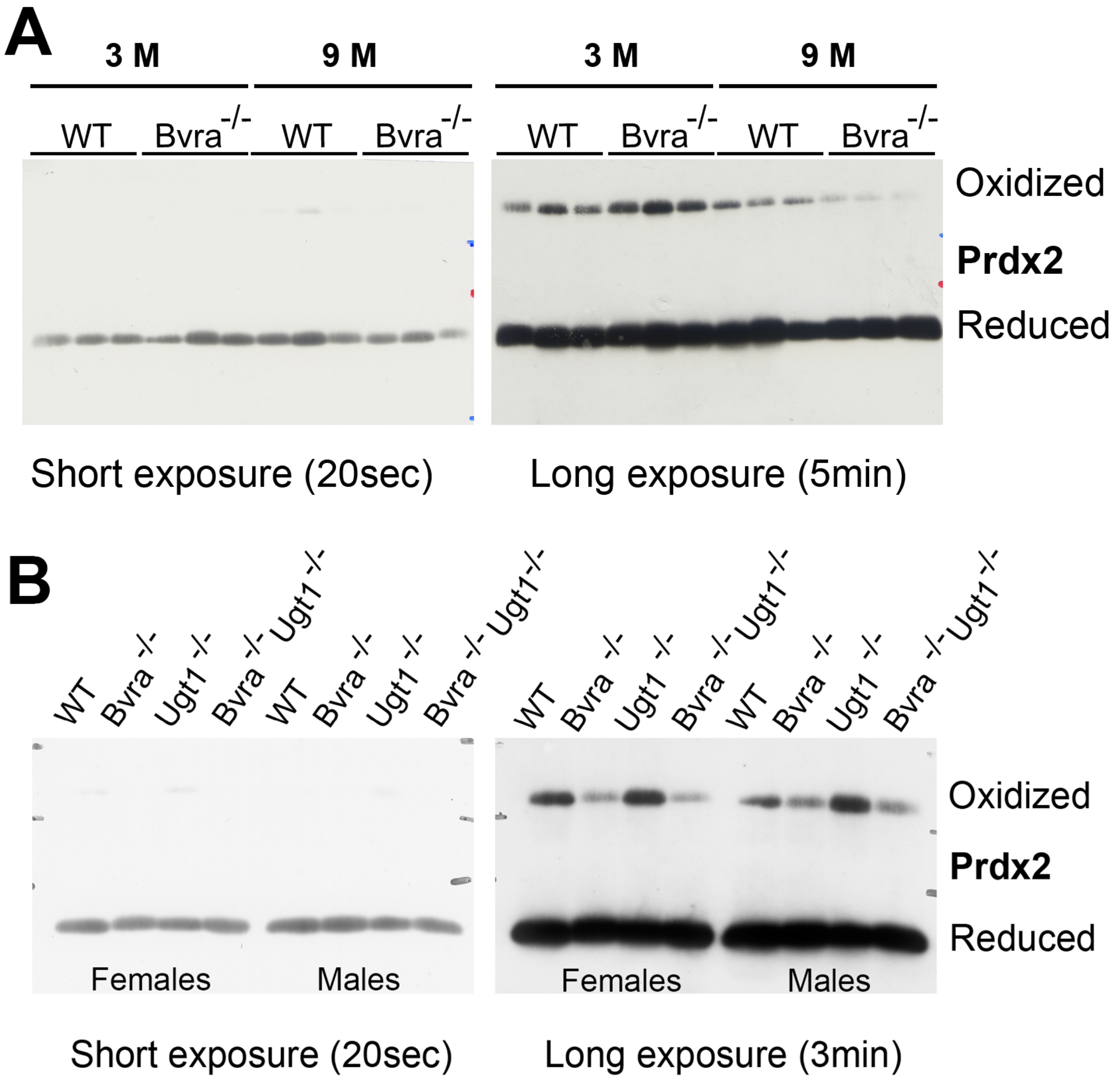

Figure S5. Western blot of peroxiredoxin in erythrocytes. Short and long exposures times of the blots shown in Figure 4C-D. A) Western blot of oxidized and reduced peroxiredoxin 2 (Prdx2 in erythrocytes of 3 and 9 months old WT and *Bvra*$^{-/-}$ mice (short and long exposure times); B) Western blot of oxidized and reduced Prdx2 in erythrocytes of 9 months old WT, *Bvra*$^{-/-}$, *Ugt1*$^{-/-}$ and *Bvra*$^{-/-}$*Ugt1*$^{-/-}$ mice (short and long exposure times).

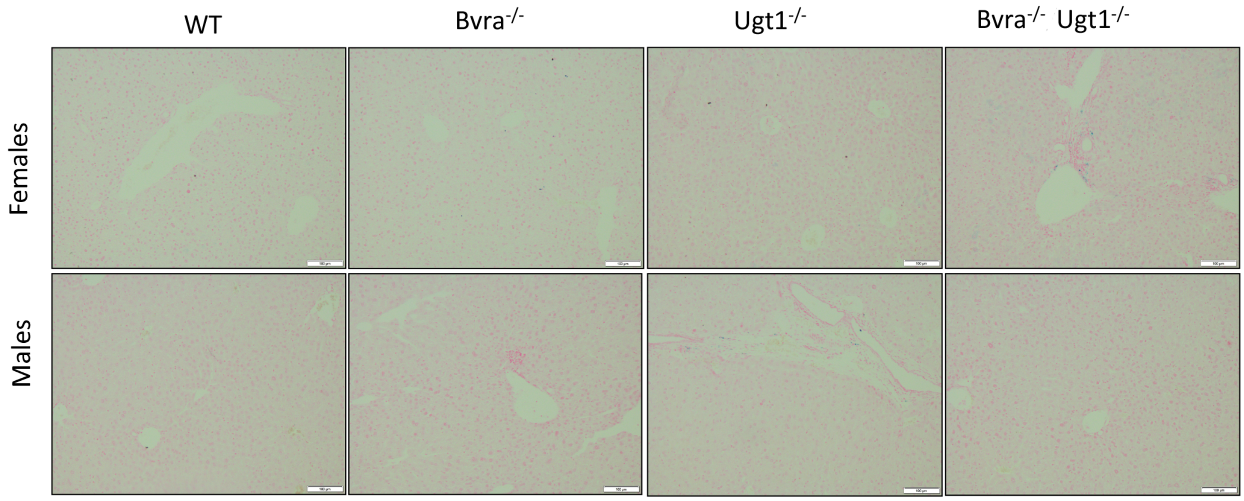

Figure S6. Prussian blue liver staining in 9 months old mice. Representative images of Prussian blue staining of liver sections of 9 months old $Bvra^{-/-}$ and $Bvra^{-/-}Ugt1^{-/-}$ mice. Scale bar 50 µm.

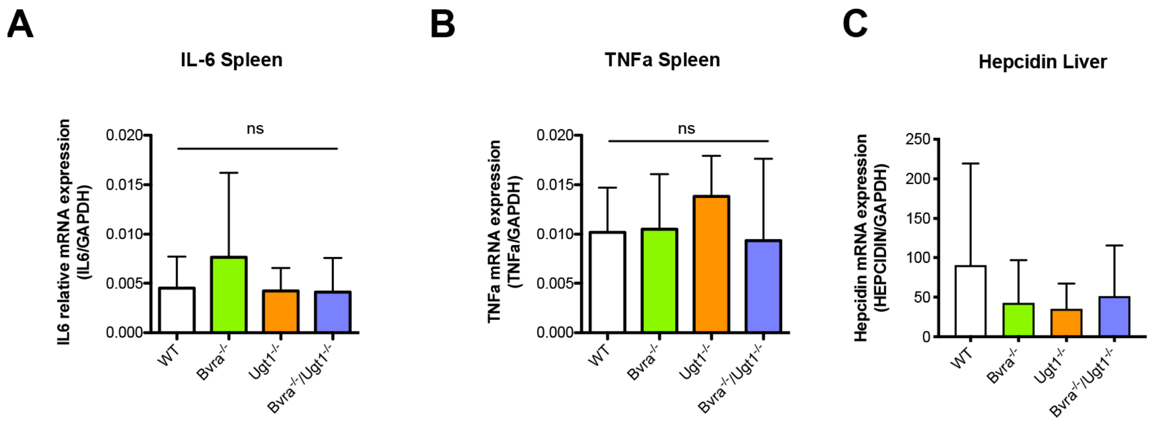

Figure S7. Relative mRNA levels of *Il6* (IL-6) and *Tnf* (TNFa) in spleen, and hepcidin in liver of 9 months old mice. A-B) Relative mRNA levels of IL-6 and TNFa are shown, relative to *Gapdh* (GAPDH) housekeeping gene; C) Relative mRNA levels of hepcidin. 1-Way Anova, P=0.3978, F=1.013, NS. Data represent mean ± SD.

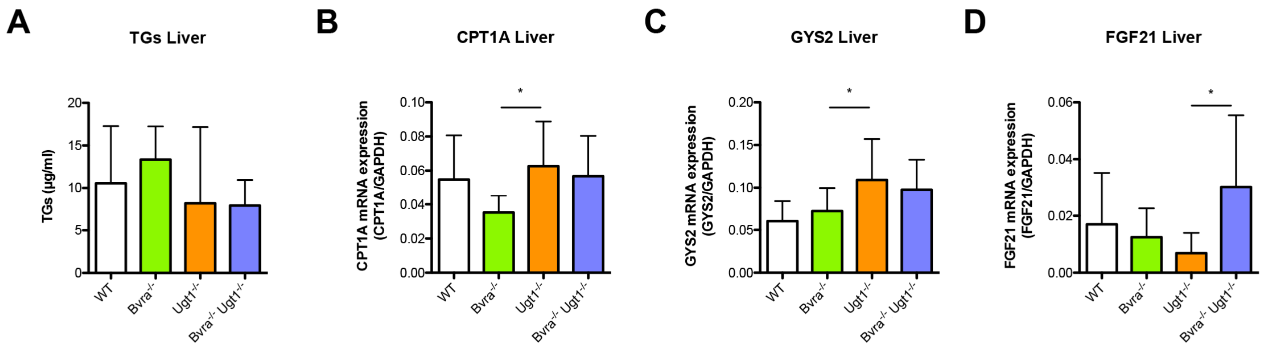

Figure S8. TG levels and PPARα responsive genes in the liver of 9 months old mice. A) Triglycerides levels (µg/ml) in liver of 9 months old animals; B-D) mRNA levels of carnitine palmitoyl transferase I (*Cpt1a*, CPT1), fibroblast growth factor 21 (*Fgf21*, FGF21), and glycogen synthase 2 (*Gys2*, GYS2), respectively. 1-way Anova: TG, P=0.3292, F=1.193, ns; *Cpt1a*, P=0.0394, F=3.085. Bonferroni post-test: WT vs. *Bvra*$^{-/-}$*Ugt1*$^{-/-}$*,* ns; *Gys2*, P=0.0144, F=4.024, Bonferroni post-test: WT vs. *Bvra*$^{-/-}$*Ugt1*$^{-/-}$*,* ns; *Fgf21*, P=0.0215,F=3.645, Bonferroni post-test: WT vs. *Bvra*$^{-/-}$*Ugt1*$^{-/-}$*,* ns.

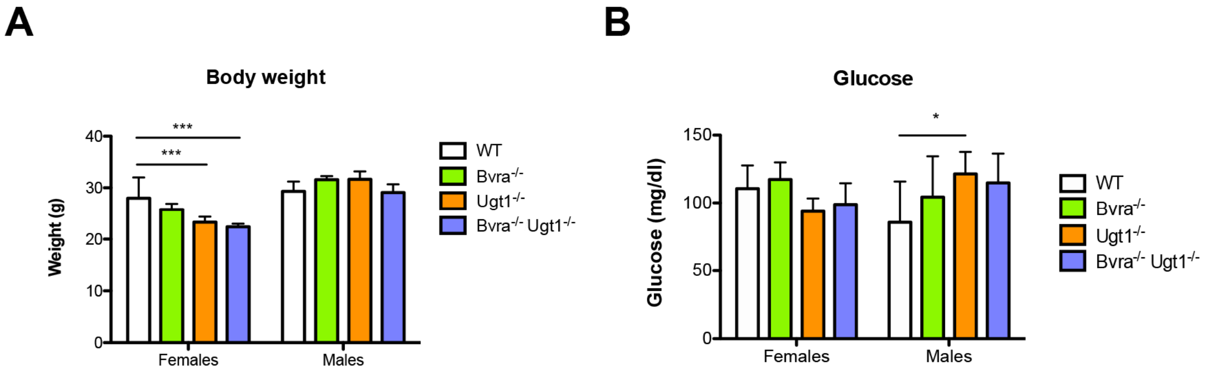

Figure S9. Body weight (A) and plasma glucose Anova (B) in 9 months old mice. Body weight (g); glucose (mg/dl). Data represent mean ± SD. 2-way ANOVA. Interaction genotype-gender ***, P=0.001; genotype, ***, P=0.002; gender, ***, P<0.0001. Female WT vs *Ugt1*$^{-/-}$, ***, P<0.001. Female *Bvra*$^{-/-}$ vs *Ugt1*$^{-/-}$, *, P<0.05. Bonferroni post-test are indicated in the corresponding panels.
